# Successful application of modified keystone flaps following skin tumor ablation

**DOI:** 10.1097/MD.0000000000017469

**Published:** 2019-10-04

**Authors:** Jiuzuo Huang, Chan Woo Kim, Xiaojun Wang, Yumo Zhao, Nanze Yu, Ru Zhao, Ming Bai, Xiao Long, Tae Hwan Park

**Affiliations:** a Department of Plastic and Reconstructive Surgery, Peking Union Medical College Hospital, Peking Union Medical College & Chinese Academy of Medical Sciences; b Department of Plastic and Reconstructive Surgery, CHA Bundang Medical Center, CHA University, Seongnam, Republic of Korea; c Peking Union Medical College, No. 9 Dongdansantiao, Dongcheng District, Beijing, People's Republic of China.

**Keywords:** flap, keystone, keystone flap, perforator, reconstruction

## Abstract

Skin cancer and precancerous skin lesions cause significant soft-tissue defects following tumor ablation. Recently, keystone flaps have gained popularity due to their simplicity, versatility, and reliability.

We evaluated the efficacy of modified keystone flaps for soft-tissue reconstruction following skin tumor ablation in 2 medical centers.

We reviewed the medical records of patients who received modified keystone flaps following the removal of skin tumors from January 2017 to December 2017. The diagnosis, site, flap size, and complications were recorded.

Forty-one modified keystone flaps were evaluated, and the wound dimensions ranged from 1 cm × 1 cm to 18 cm × 9.5 cm, with an average size of 9.8 cm × 6.4 cm. With our selection strategy, specific modified keystone flaps were designed for the soft-tissue defects. The flap dimensions ranged from 2.2 cm × 1 cm to 26 cm × 10 cm, with an average size of 14.3 cm × 7.5 cm. Two patients developed minor wound dehiscence (4.9%), and 1 patient developed partial flap loss (2.4%), but all of these patients healed after local wound care without the need for surgical intervention.

Our selection strategy for modified keystone flaps is a feasible and reliable option for reconstruction following skin tumor excision.

## 1 Introduction

The keystone perforator island flap (KPIF) is a multiperforator advancement flap that was first reported by Behan in 2003.^[1]^ The keystone flap is an excellent new tool for reconstructive surgery and has wide clinical applications. These flaps have a safe flap harvest technique, a reliable blood supply, minimal donor site morbidity, and a simple dissection process that obviates microsurgical techniques.^[2]^

**Figure 1:**
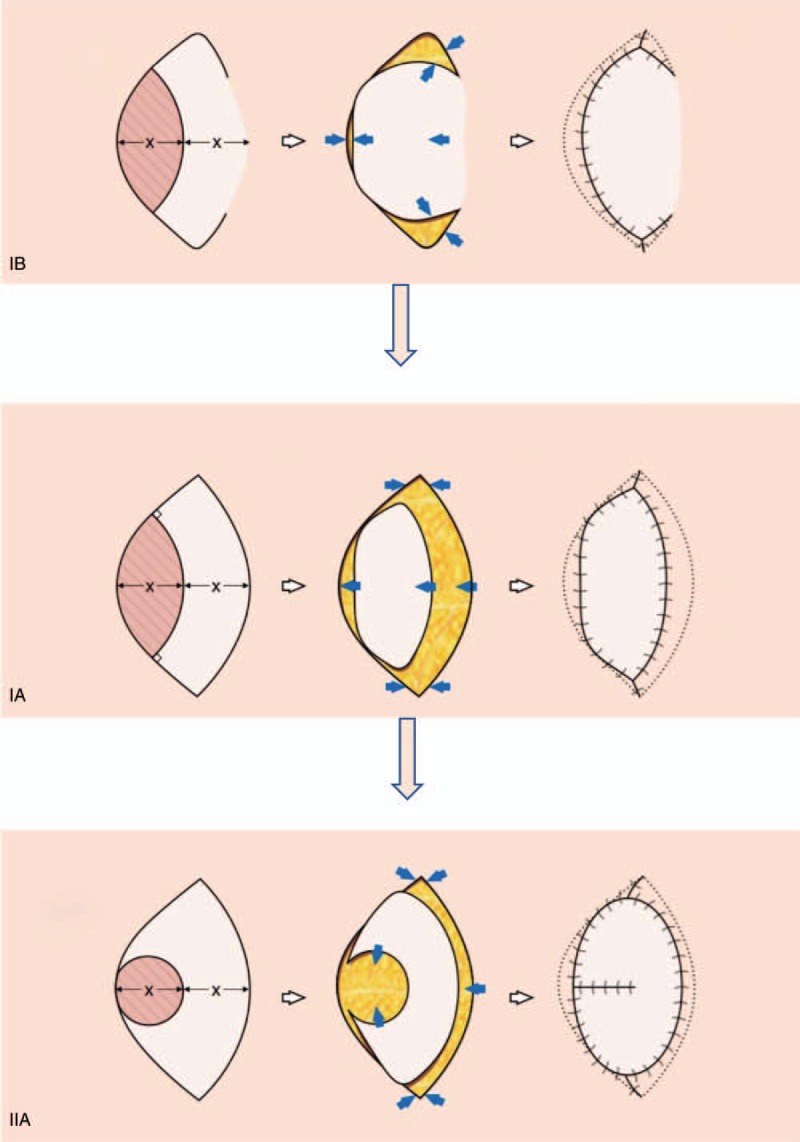
Schematic diagram of the keystone flaps. Type Ia flap: cover the defect with a standard keystone perforator island flap without a skin bridge. Type Ib flap: keystone flap with a skin bridge. Type IIa flap: keystone perforator island flap without a skin bridge, and both arms are elevated and transposed to cover the defect. Our selection strategy is as follows: first try a type Ib flap, then try a type Ia flap, and take a type IIa flap as the last resort.

KPIFs have a curvilinear trapezoidal shape that represents the architectural shape of the keystone in Roman arches and are designed over dermatomal segments to have a flap width to elliptical defect ratio of 1:1 (Fig. 1A). Since the first introduction of KPIFs, several modifications have been made for the effective reconstruction of soft-tissue defects of various sizes and shapes.^[2–6]^ These modifications include maintaining a skin bridge,^[3]^ partial undermining,^[3]^ and folding the flap into an “omega.”^[4]^ However, no selection strategy exists to determine the most suitable modified keystone flap for a specific defect.

This study aimed to introduce our reconstruction experience after skin tumor resection and our selection strategy for modified keystone flaps based on our clinical experience.

## 2 Materials and methods

We obtained written informed consent from all patients. The study protocol conformed to the ethical guidelines of the 1975 Declaration of Helsinki.

This multicenter study, with 2 medical centers, was conducted from January to December 2017. We retrospectively reviewed the charts of patients who received keystone flaps following skin tumor resection. We recorded each patient's age, sex, diagnosis, smoking status, comorbidities (e.g., diabetics and hypertension), pathological diagnosis, surgical site, surgical procedure, defect dimensions, flap dimensions, and complications.

### 2.1 Surgical technique

In this study, we adopted 3 different types of modified keystone flaps for soft-tissue reconstruction. A schematic diagram of the surgical technique is provided in Fig. 1.

The defect size was measured preoperatively; the defect length was the longest dimension, and the defect width was the dimension perpendicular to the length. Each lesion was sufficiently resected or debrided, and elliptical or round wounds were created depending on the initial wound. Tissues adjacent to the defect were manipulated, and the side with greater laxity was used for flap design. Then, a longitudinal curvilinear keystone flap was designed, and the limbs of the flap were drawn 90° to the longitudinal axis of the defect. The width of the flap was equal to the width of the wound.

After an incision was circumferentially made into the skin and down to the fascia, a type Ia flap was harvested with careful dissection and minimal undermining to avoid compromising the perforator vascular supply. A partial or circumferential incision into the deep fascia could be made to improve the motility of the flap for better tissue approximation. The flap was advanced in place over the defect, and the first suture was placed in the center of the flap with maximal tension. Perpendicular to the flap advancement, the 2 peripheral borders were advanced in a V-Y fashion. The incision was closed with single interrupted or horizontal mattress sutures, and a drain was inserted if necessary.

In contrast to original keystone flaps, type Ib flaps do not completely create a skin island. A skin bridge is left intact along the greater arc of the flap, which not only affords additional vascularity but also preserves the subdermal lymphatics to reduce the risk of pin cushioning that often occurs in fully islanded flaps. A lateral fasciotomy can be performed with a pair of suitably sized scissors inserted through the limited incision on the lateral arc. This modification effectively shortens the overall flap scar.

The type IIa flap uses the tissue adjacent to the defect and both arms to cover the defect. The skin flaps adjacent to the defect were elevated completely off the deep fascia to facilitate closure, similar to how a fortune cookie is closed.^[7]^

Our selection strategy is as follows: first try a type Ib flap while preserving a skin bridge; if the type Ib flap cannot effectively cover the defect, then try a type Ia flap with an incision that covers the full length of the greater arc; if the type Ia flap still fails, then try a type IIa flap and transposition of both arms of the keystone flap to cover the defect.

## 3 Results

**Figure 2:**
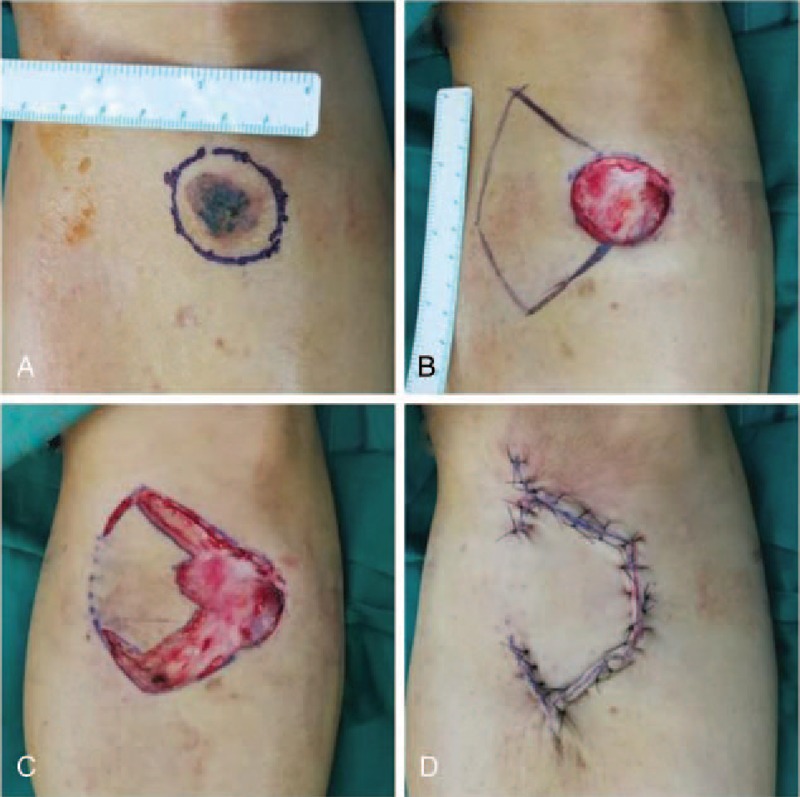
(A) A 61-year-old man with squamous cell carcinoma of the left leg. (B) Intraoperative view of a defect measuring 3 cm × 3 cm after wide excision of the cancer. (C) Intraoperative view after incising a keystone flap with a skin bridge (type Ib). (D) Immediate postoperative result of a type Ib keystone flap.

**Figure 3:**
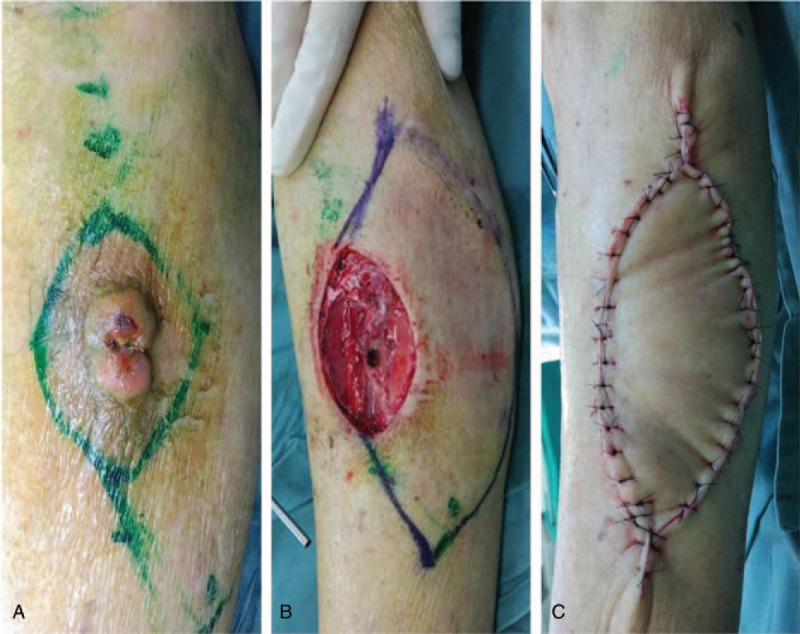
(A) A 77-year-old man with squamous cell carcinoma of the left leg. (B) Intraoperative view of a defect measuring 7 cm × 4 cm after wide excision of the cancer. (C) Immediate postoperative result of a type Ia keystone flap.

**Figure 4:**
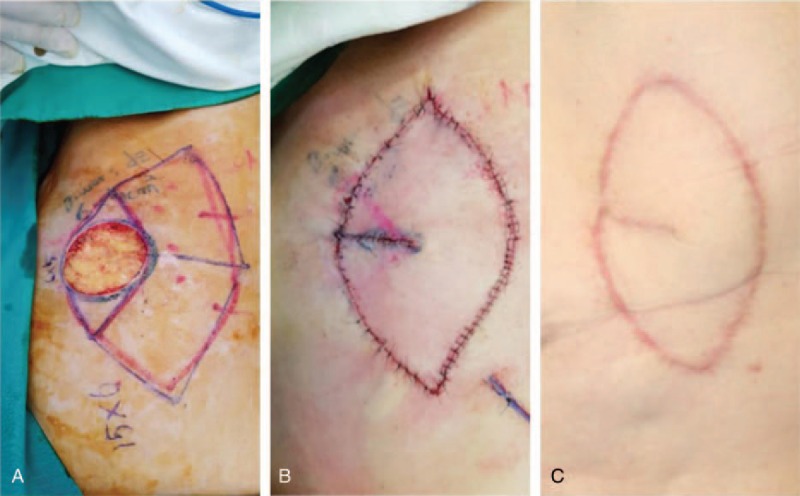
A 67-year-old woman with Bowen disease on her left flank. (A) Intraoperative view of a defect measuring 6 cm × 5 cm after wide excision of the tumor. (B) Immediate postoperative result of a type IIa keystone flap. (C) Postoperative view 5 months later.

Forty-one modified keystone flaps were performed in total. The baseline patient demographics are listed in Table 1. In total, 17 patients had basal cell carcinoma, 14 patients had squamous cell carcinoma, 6 patients had soft-tissue sarcoma, and 4 patients had keloids. For low-risk basal and squamous cell carcinomas, a clinical margin of 5 mm was chosen. For high-risk basal and squamous cell carcinomas, a clinical margin of 1 cm was chosen. For soft-tissue sarcomas, a clinical margin of 3 cm was chosen. Negative tumor resection margins were achieved according to intraoperative frozen section pathology. The dimension of the wound defects were between 1 cm × 1 cm and 18 cm × 9.5 cm, with an average size of 9.8 cm × 6.4 cm. The size of the keystone flaps was between 2.2 cm × 1 cm and 26 cm × 10 cm, with an average size of 14.3 cm × 7.5 cm. A total of 32 defects (78.0%) were repaired with type Ia keystone flaps, 5 defects (12.2%) with type Ib flaps, and 4 defects (9.8%) with type IIa flaps. Some typical cases are listed in Figs. 2–4.

**Table 1:**
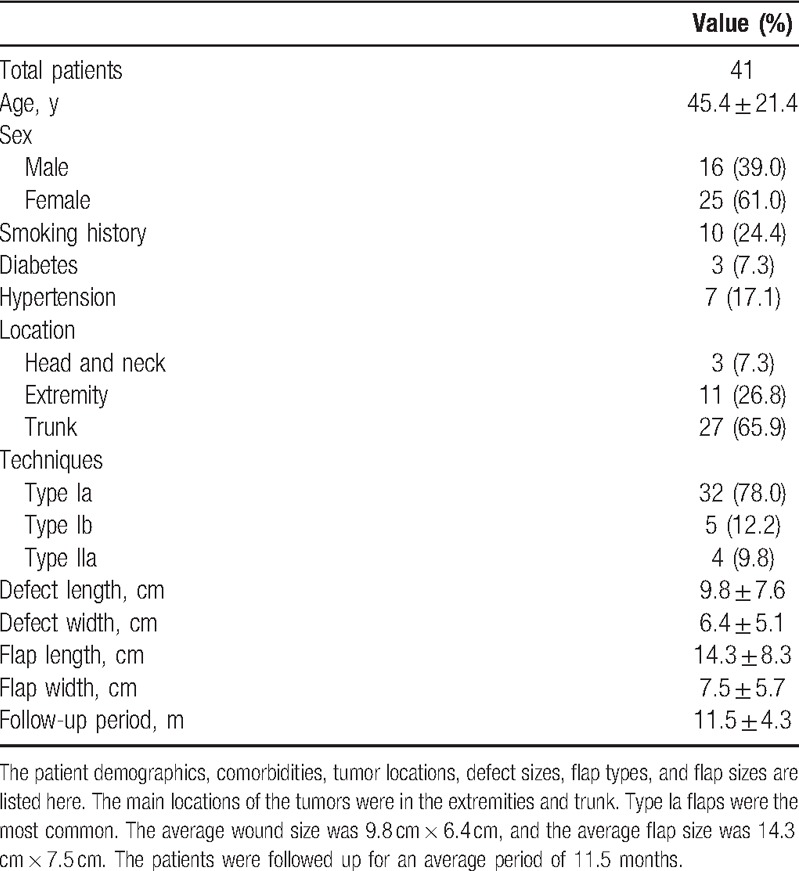
Patient demographics and wound/flap descriptions.

The flaps healed smoothly without any major complications, and the postoperative pain was minimal. Two patients developed minor wound dehiscence (4.9%), and 1 patient developed partial flap loss (2.4%), but all of these patients healed after local wound care without the need for additional operations. The surgery-related details are listed in Tables 1–3.

**Table 2:**
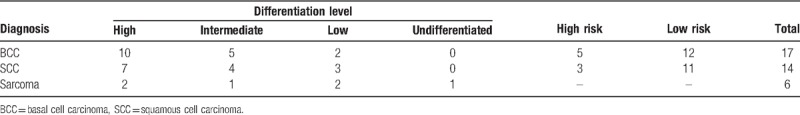
Oncological information.

**Table 3:**
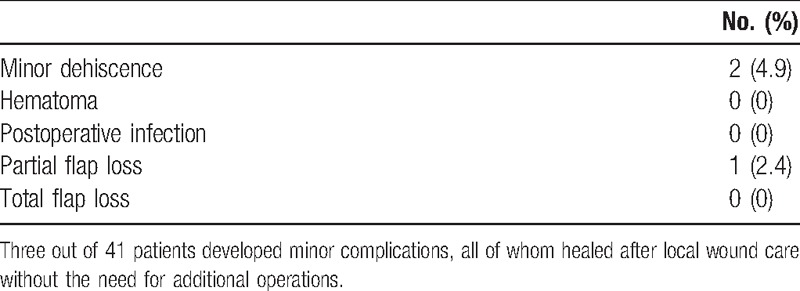
Complications.

The patients were closely followed up, and 1 patient with undifferentiated pleomorphic sarcoma in the shoulder region developed recurrence 13 months after the previous operation with a keystone flap. The patient underwent radical resection and pedicled latissimus dorsi flap reconstruction.

## 4 Discussion

The clinical applications of the keystone flap have evolved over the past decade^[8,9]^ to cover large defects in the trunk,^[9,10]^ limbs,^[11–13]^ perineum,^[14,15]^ and head and neck.^[16–19]^ In this study, 3 different types of modified keystone flaps were used to repair soft-tissue defects following skin tumor ablation. The selection strategy we applied for these 41 keystone flaps is feasible and has a high success rate for reconstruction. The greatest advantage of the keystone flap for skin tumor reconstruction is the simplicity of the flap, which can be performed in 1 stage without any donor site morbidity, thus enabling reconstruction with similar tissues. The notable other benefit of keystone flaps is the redistribution of tissue laxity and tension over the entire flap to cover the defect.^[20,21]^ Because the above-mentioned reasons allow aesthetically pleasing results to be achieved, most patients are highly satisfied with the final outcome regardless of the type of modification selected.

Additionally, the use of adjacent tissue can help surgeons widely excise skin cancer with sufficient margins either laterally or deeply as well as reduce the operative time, obviate the need to dissect tiny perforators, and offer a reconstruction approach with a high success rate, relatively pain-free recovery, minimal donor site morbidity, and early mobilization. Compared with microsurgical free flaps, keystone flaps do not need tedious dissection or vascular anastomosis with a surgical microscope, have less donor site morbidity, and have a much higher success rate.

Since the introduction of keystone flaps, several modifications have been made to the original keystone flaps to effectively reconstruct soft-tissue defects of various sizes and shapes.^[2–6]^ These modifications include maintaining a skin bridge,^[3]^ partial undermining,^[3]^ and folding the flap into an “omega.”^[4]^ However, no one single flap or modification can be applied for all situations. The surgeon should apply a selection strategy to choose the correct modified keystone flap for each specific defect. However, no such strategy for selecting the specific modified keystone flap for a given defect exists.

The advantages and limitations of traditional keystone flaps must be understood before creating a selection strategy. One of the disadvantages of the traditional keystone flap is the long skin incision. Type Ib flaps preserve a skin bridge along the greater arc, thus effectively shortening the length of the skin incision and theoretically, improving the venous and lymphatic drainage. Incising the fascia under the tunneled skin bridge allows for advancement and prevents deep structure shearing.^[22]^ On the other hand, sometimes traditional keystone flaps cannot repair the defect via only tissue advancement. Created by the transposition of both arms of the keystone flap, type IIa flaps could improve the ability for reconstruction. We believe that type IIa keystone flaps are an oncologically effective option in terms of cancer surveillance after complete wound healing because the linear wound in the center of the flap is closest to the primary cancerous lesion. Therefore, when recurrence develops, we can easily anticipate which area should be further excised.

Attempting to approximate the wound edges with a pair of skin hooks or towel clips after excising the lesion and/or incising the flap can be useful for gauging the extent of the reconstruction required. This simple maneuver allows the surgeon to gauge the tension that needs to be overcome and adjust the operative plan according to the selection strategy.

No major complications occurred in our case series, indicating that our selection strategy for keystone flaps is feasible. The scarring in the reconstruction site was minimal, and the color match was excellent. All patients were satisfied with the final appearance and function of the flaps.

Our study includes only 3 types of modified keystone flaps, type Ia, type Ib, and type IIa. However, Lee et al^[6]^ designed 6 types of keystone flaps. In their study, type Ia, type Ib, and type IIa accounted for 15%, 23%, and 54% of all flaps, respectively, or 92% of all flaps. The other 3 types (IIb, IIIa, and IIIb) accounted for only 8% of all flaps. Therefore, our study included the 3 most common and highly representative types of modified keystone flaps. The selection strategy for these 3 types of keystone flaps is suitable for reconstruction with keystone flaps.

### 4.1 Limitations

This is a retrospective study with a limited number of cases. To further verify the effectiveness of our selection strategy for keystone flaps, a prospective study with more patients and various defect sizes and locations should be conducted. The tumor borders were confirmed by frozen sections, which is not as accurate as Mohs micrographic surgery. Since the keystone flap, which is a local flap, utilizes the adjacent tissue to repair the defect after tumor resection, a certain limitation exists for the oncological margins of resection, including the deep margin. Tumor surveillance and patient follow-up are of great importance for patients after keystone flap reconstruction.

## 5 Conclusion

In conclusion, with our selection strategy for modified keystone flaps, various soft-tissue defects can be successfully repaired. Our selection strategy is feasible and reliable for soft-tissue reconstruction with keystone flaps.

## Author contributions

**Conceptualization:** Jiuzuo Huang, Ru Zhao, Nanze Yu, Xiao Long, Tae Hwan Park.

**Data curation:** Jiuzuo Huang, Chan Woo Kim, Nanze Yu, Xiao Long.

**Formal analysis:** Chan Woo Kim, Yumo Zhao, Nanze Yu, Xiao Long.

**Funding acquisition:** Xiao Long.

**Investigation:** Jiuzuo Huang, Xiaojun Wang, Ru Zhao, Ming Bai, Xiao Long, Tae Hwan Park.

**Methodology:** Jiuzuo Huang, Chan Woo Kim, Xiaojun Wang, Ru Zhao, Ming Bai, Xiao Long, Tae Hwan Park.

**Project administration:** Ming Bai, Xiao Long.

**Resources:** Xiao Long.

**Software:** Yumo Zhao.

**Supervision:** Xiaojun Wang, Ru Zhao, Tae Hwan Park.

**Validation:** Ming Bai.

**Writing – original draft:** Jiuzuo Huang, Chan Woo Kim, Yumo Zhao, Ru Zhao, Ming Bai, Nanze Yu, Xiao Long, Tae Hwan Park.

**Writing – review & editing:** Xiaojun Wang, Ru Zhao, Ming Bai, Xiao Long, Tae Hwan Park.
